# Orthostatic Challenge Shifts the Hemostatic System of Patients Recovered from Stroke toward Hypercoagulability

**DOI:** 10.3389/fphys.2017.00012

**Published:** 2017-02-07

**Authors:** Gerhard Cvirn, Markus Kneihsl, Christine Rossmann, Margret Paar, Thomas Gattringer, Axel Schlagenhauf, Bettina Leschnik, Martin Koestenberger, Erwin Tafeit, Gilbert Reibnegger, Irhad Trozic, Andreas Rössler, Franz Fazekas, Nandu Goswami

**Affiliations:** ^1^Institute of Physiological Chemistry, Medical University of GrazGraz, Austria; ^2^Department of Neurology, Medical University of GrazGraz, Austria; ^3^Department of Pediatrics, Medical University of GrazGraz, Austria; ^4^Gravitational Physiology, Aging and Medicine Research Unit, Institute of Physiology, Medical University of GrazGraz, Austria

**Keywords:** thrombin, thrombosis, tissue factor, stroke/prevention, standing, orthostasis, coagulation, thrombo-embolism

## Abstract

**Aims:** The objective of our study was to assess the effects of orthostatic challenge on the coagulation system in patients with a history of thromboembolic events and to assess how they compared with age-matched healthy controls.

**Methods:** Twenty-two patients with histories of ischemic stroke and 22 healthy age-matched controls performed a sit-to-stand test. Blood was collected prior to- and at the end of- standing in the upright position for 6 min. Hemostatic profiling was performed by determining thrombelastometry and calibrated automated thrombogram values, indices of thrombin generation, standard coagulation times, markers of endothelial activation, plasma levels of coagulation factors and copeptin, and hematocrit.

**Results:** Orthostatic challenge caused a significant endothelial and coagulation activation in patients (Group 1) and healthy controls (Group 2): Plasma levels of prothrombin fragment F1+2 were increased by approximately 35% and thrombin/antithrombin-complex (TAT) increased 5-fold. Several coagulation variables were significantly altered in Group 1 but not in Group 2: Coagulation times (CTs) were significantly shortened and alpha angles, peak rate of thrombin generation (VELINDEX), tissue factor (TF) and copeptin plasma levels were significantly increased (comparison between standing and baseline). Moreover, the shortening of CTs and the rise of copeptin plasma levels were significantly higher in Group 1 vs. Group 2 (comparison between groups).

**Conclusion:** The coagulation system of patients with a history of ischemic stroke can be more easily shifted toward a hypercoagulable state than that of healthy controls. Attentive and long-term anticoagulant treatment is essential to keep patients from recurrence of vascular events.

## Introduction

Several studies have reported that an orthostatic challenge activates the coagulation system. Both active and passive standing are associated with (i) pooling of blood in the legs, (ii) increased transmural pressure, (iii) increased shear stress, as well as (iv) endothelial activation in the lower extremities, associated with (v) release of procoagulant glycoproteins, e.g., Tissue Factor (TF) (Masoud et al., 2008; Cvirn et al., 2012; Haider et al., 2013). All these studies have been performed in healthy young controls. Despite coagulation activation, no cases of thrombosis have been reported in these persons. It, therefore, appears that an orthostatic challenge, induced by either active or passive standing, does not provoke thromboembolic events in healthy controls. However, orthostatic challenge-induced coagulation activation might constitute a problem for patients at an already elevated risk for vascular events, for example, ischemic stroke.

We, therefore, wanted to investigate the influence of orthostatic challenge on the coagulation system in patients with a history of ischemic stroke. In our study, 22 older patients, who had recovered from ischemic stroke, performed a sit-to-stand test, a suitable model for orthostatic challenge. Twenty-two healthy age-matched controls served as controls.

Hemostatic profiling was carried out in both whole blood and platelet poor plasma samples. The clot formation process was monitored using TF-triggered thrombelastometry. Thrombin generation was assessed by means of calibrated automated thrombography (CAT), and by the determination of plasma levels of prothrombin fragment 1+2 (F1+2) and thrombin-antithrombin complexes (TAT). For further assessment of orthostatic challenge-induced coagulation activation, we measured prothrombin times (PT), activated partial thromboplastin times (APTT), plasma levels of FII, FVII, FVIII, protein C, and protein S. In order to evaluate orthostatic challenge-induced endothelial activation, we determined plasma levels of TF and of tissue-plasminogen activator as well as of nitric oxide. Since nitric oxide has a very short half-life, it is very difficult to determine nitric oxide content in whole blood samples. We therefore measured nitrite + nitrate levels, the major stable metabolites of nitric oxide, which can be quantitatively determined (Romitelli et al., 2007).

It has been shown that standing is associated with hemoconcentration due to the transfer of intravascular fluid from the blood to the surrounding tissue (Hinghofer-Szalkay and Moser, 1986; Jacob et al., 2005). We therefore determined hematocrit values in pre- and post-orthostatic challenge blood samples to compare the hemoconcentration effect of orthostatic loading (standing) in both groups. Plasma volume changes were calculated from hematocrit, as described by Masoud et al. (2010).

Moreover, copeptin levels have been shown to be sensitive surrogate markers for the release of arginine-vasopressin, which is associated with orthostatic challenge (Goldsmith, 1989; Choi et al., 2015). We therefore determined levels of copeptin in pre- and post-orthostatic challenge plasma samples of the two groups in order to evaluate the orthostatic challenge-response on the hormonal levels.

## Materials and methods

### Subjects and experimental design

Twenty-two patients who had recovered from ischemic stroke and 22 healthy controls performed a sit-to-stand test. The patients had recovered from (their first) mild stroke syndromes (NIHSS: 1–3), which occurred not longer than 12 months before the measurements that were carried out in this study. The patients and healthy controls characteristics are shown in Table 1.

**Table 1:** **Participants' characteristics**.

	**All patients (*n* = 44)**	**Stroke group (*n* = 22, Group 1)**	**Control group (*n* = 22, Group 2)**	***P*-value (Group 1 vs. Group 2)**
Gender, female/male	19/25	7/15	12/10	n.s.
Age (years), mean ± SD	63.8 ± 7.0	62.4 ± 6.7	65.2 ± 7.1	n.s.
Body mass index, mean ± SD, kg/m^2^	28 ± 7	27 ± 5	28 ± 5	n.s.
**ANTICOAGULANT THERAPY**				
ASA (100 mg), n (%)	18 (41)	15 (68)	3 (14)	0.0003
Clopidogrel (75 mg), n (%)	6 (14)	6 (27)	0 (0)	0.0110
Antidiabetic therapy, n (%)	2 (5)	1 (4)	1 (4)	n.s.
Antihypertensive therapy, n (%)	23 (52)	16 (73)	7 (32)	0.0074
Previous vascular events, n (%) (venous thromboembolism, MCI)	2 (5)	2 (10)	0 (0)	<0.0001
**VASCULAR RISK FACTORS**				
Arterial hypertension, n (%)	24 (55)	18 (82)	6 (27)	0.0003
Dyslipidemia, n (%)	21 (48)	16 (73)	5 (23)	0.0011
Diabetes mellitus, n (%)	2 (5)	1 (5)	1 (5)	n.s.
Nicotine usage	4 (9)	3 (14)	1 (5)	n.s.
Obesity > grade 1, n (%)	4 (9)	3 (14)	1 (5)	n.s.
Atrial fibrillation, n (%)	4 (9)	4 (18)	0 (0)	n.s.
NIHSS at baseline, median (range)	0 (0–3)	1 (0–3)	0 (0)	<0.0001

*A total of 44 individuals performed a sit-to-stand test. Group 1 comprised of 22 older patients who had recovered from ischemic stroke, 22 healthy age-matched controls served as controls (Group 2). P-values were calculated by means of the Fischer's exact test. ASA, acetylsalicylic acid; MCI, myocardial infarction; NIHSS, National Institutes of Health Stroke Scale*.

After 5 min of sitting still, a blood sample (*baseline*) was collected from the antecubital vein. Subsequently, test subjects were assisted into the upright positon for 6 min; another blood sample was then withdrawn from the vein (*standing sample*). While standing, test subjects kept their eyes open and did not alter foot placement.

Experiments were carried out in a room with minimal ambient noise. The room temperature was maintained between 23 and 25°C and the experiments were carried out between 7 and 11 a.m. All control subjects underwent basic neurological assessment prior to our investigations.

The Ethics Committee of the Medical University of Graz, Austria approved this study (EK-Nr. 25-551 ex 12/13). Both patients and healthy controls provided informed written consent before taking part in the study.

### Blood sampling

#### Baseline samples

Twelve milliliters of venous blood were collected in pre-citrated Vacuette® tubes, which contained 3.8% sodium citrate (Greiner Bio-one GmbH, Kremsmünster, Austria). Additionally, 4 mL of venous blood was collected into EDTA- and aprotinin-treated tubes to determine copeptin concentrations. Haematocrit and thrombelastometry measurements were performed in citrated whole blood samples. Subsequently, the remaining whole blood underwent centrifugation at 2000 g for 20 min in order to prepare platelet poor plasma samples. The remaining measurements were performed in platelet poor plasma samples.

#### Standing samples

After the subjects spent 6 min in an upright position, venous blood samples were withdrawn in the same way- and measurements performed- as described for the baseline samples.

### TF-triggered thrombelastometry assay

The clot formation process was monitored using the thrombelastometry coagulation analyser (ROTEM®05, Matel Medizintechnik, Austria). The following were measured: *CT (Coagulation time)*: which is time from adding trigger to formation of initial fibrin formation; *Clot formation time (CFT)*: which is the time taken until the amplitude reaches 20 mm; *Maximum clot firmness (MCF)*: which reflects clot stability; *Alpha angle*: which indicates the velocity of fibrin built-up and cross-linking. For this procedure, the volume of blood sample required was 340 μL and clot formation was initiated by adding 40 μL of “trigger solution” (containing 0.35 pmol/L TF and 3 mmol/L CaCl_2_, final concentration) to 300 μL of citrated whole blood (Sørensen et al., 2003).

### Thrombin generation assessments via the automated fluorogenic measurements

CAT obtained from Thrombinoscope BV (Maastricht, the Netherlands) was used to monitor Thrombin generation curves (Hemker et al., 2003). Following laboratory values were determined: *Lag Time:* which is the time preceding the thrombin burst; *Endogenous thrombin potential (ETP)* and *peak height (Peak)*: Either of these reflect the amount of thrombin being generated; *ttPeak*: time to peak; *VELINDEX [peak thrombin/(peak time* − *lag time)]*: the peak rate of thrombin formation; *StartTail*: the time at which no free thrombin is measurable. Low amounts of trigger (5 pmol/L of TF) were used for sensitive detection of thrombin formation.

### Standard laboratory tests

BM/Hitachi 917 (Roche, Austria) was used to determine the following: PT: expressed as International Normalized Ratio, INR; APTT; plasma levels of FII; FVII; FVIII; protein C; and protein S. ELISA kits (Behring Diagnostics GmbH, Marburg, Germany) were used to measure F 1+2 levels in the plasma and TAT. TF plasma levels were determined by applying ACTICHROME Tissue Factor ELISA and tissue-plasminogen activator concentrations were determined using the IMUBIND tissue-plasminogen activator ELISA kit (American Diagnostica, Pfungstadt, Germany).

### Haematocrit and blood cell counts

Sysmex KX-21 N Automated Haematology Analyzer from Sysmex (Illinois, USA) was used to determine hematocrit and blood cell counts.

### Nitrite+nitrate plasma levels

Two hundred microliters of plasma (baseline and standing samples) were diluted with double-distilled water (1:2, vol/vol) and loaded on pre-conditioned anion exchange columns (Chromabond SB, Macherey-Nagel, Düren, Germany). After a washing step with double-distilled water nitrite and nitrate were eluted with 1 mL 0.5 mol/L sodium chloride. In the eluates, nitrite and nitrate were determined simultaneously by means of HPLC analysis according to a previously published method (Romitelli et al., 2007) but with some modifications. Briefly, the HPLC consisted of a L-2200 autosampler, two L-2130 HTA pumps, and a L-2450 diode array detector (all: VWR Hitachi, VWR, Vienna, Austria). Separation was performed on a Hypersil ODS column (5 μm; 250 × 4 mm I.D.) with 10.0 min isocratic elution (buffer A: 0.1 mol/L NaH_2_PO_4_, pH = 5.5, containing 5.9 mmol/L tetrabutylammonium hydrogensulphate) followed by a linear gradient to 20% buffer B (buffer B: 0.1 mol/L NaH_2_PO4, pH = 5.5, containing 5.9 mmol/L tetrabutylammonium hydrogensulphate/acetonitrile, 3:1, vol/vol) within another 10 min. The injection volume of standard and sample solutions was 40 μL. The absorbance at 205 nm was recorded. Data acquisition and subsequent analysis was done with the EZchrom Elite (VWR) program. Retention time was ~7.80 min for nitrite and ~14.5 min for nitrate.

### Concentration of copeptin

Levels of copeptin were determined in heparinized plasma samples by means of a sandwich enzyme immunoassay from Wuhan USCN Business Co., Ldt (Houston, USA).

### Statistics

**Figure 1:**
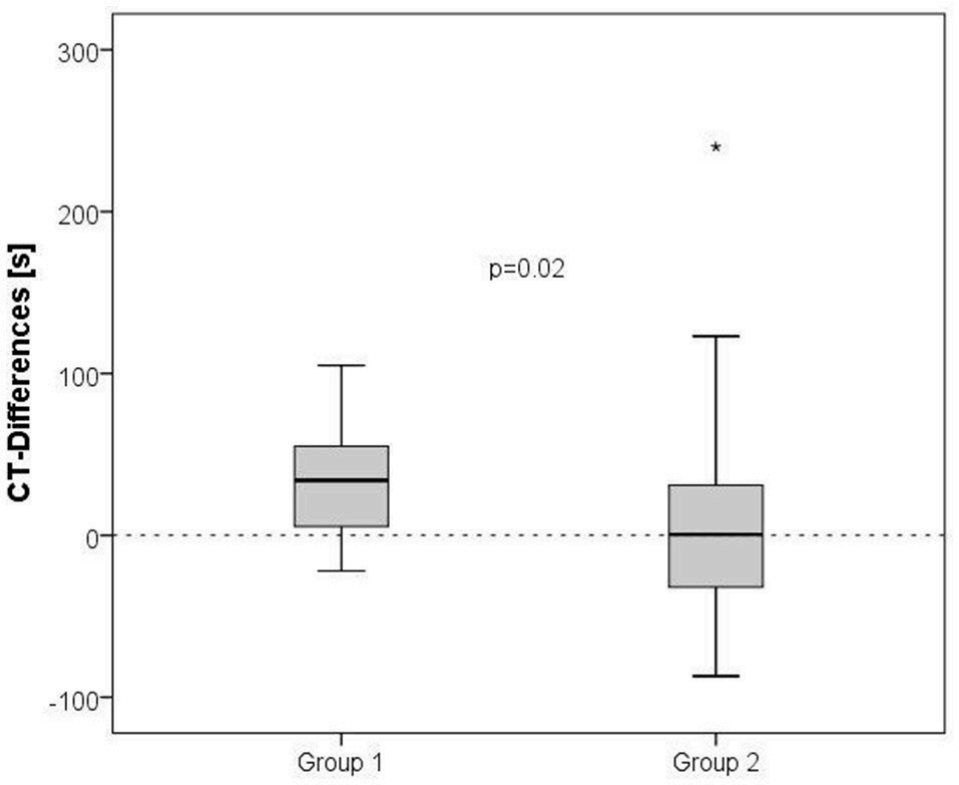
**Orthostatic challenge-induced shortening of thrombelastometry-derived CTs in patients recovered from stroke (Group 1) vs. healthy, age-matched controls (Group 2)**. A boxplot of CT-differences for the two groups is shown. *P*-values were calculated by means of the Mann-Whitney *U*-test. “^*^” is an outlier.

**Figure 2:**
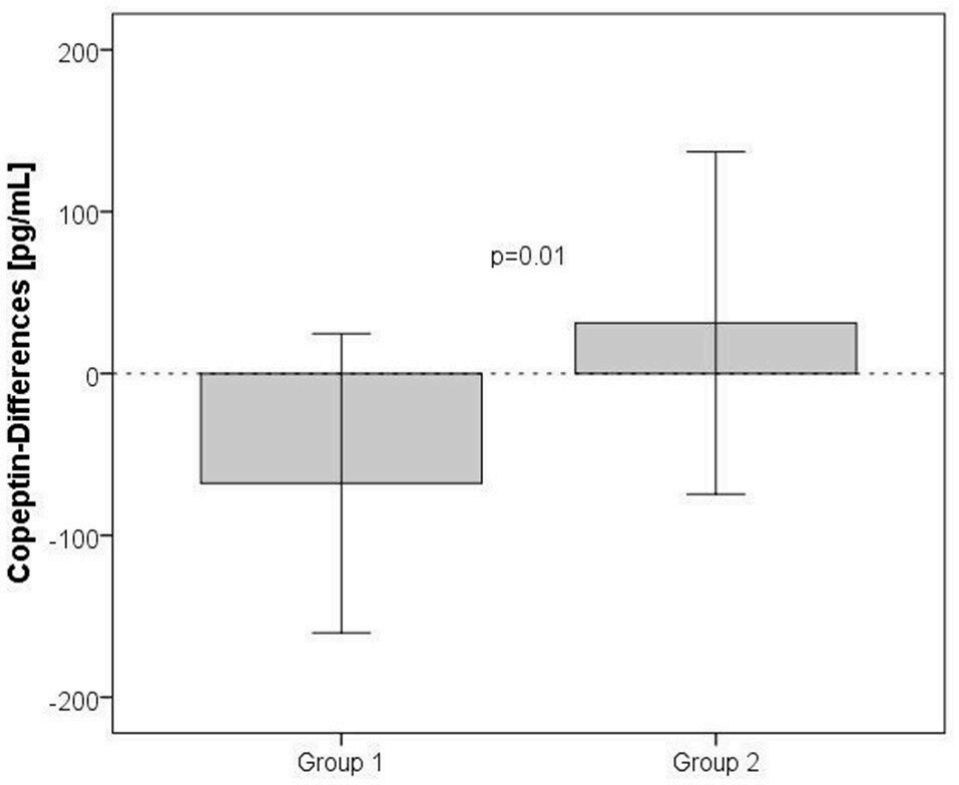
**Orthostatic challenge-induced rise of copeptin plasma levels in patients recovered from stroke (Group 1) vs. healthy, age-matched controls (Group 2)**. A bar diagram of copeptin-differences for the two groups presenting means ± SDs is shown. *P*-values were calculated by means of the *t*-test for independent samples.

IBM SPSS Statistics 23 (IBM, New York, USA) program was used for statistical analyses. Differences in Table 1 between group 1 (patients recovered from stroke) and group 2 (healthy, age-matched controls) were determined by means of the Fischer's exact test. The variables that were normally distributed were tested by the Kolmogorov-Smirnov test and the Shapiro-Wilk test. Values of *p* < 0.05 were considered as significant deviations from the normal distribution. The median and the interquartile range were calculated and presented in Tables 2–**4** since most variables were not normally distributed. Furthermore, to investigate the significance of differences before and after orthostatic challenge, the Wilcoxon test was used for not normally distributed variables. If variables were normally distributed, a *T*-test for paired samples was applied. Finally, to compare the changes between the patients and age-matched healthy controls (Figures 1, 2), the difference between the standing and the baseline value was calculated for each variable (for example: CT-difference = CT-baseline − CT-standing). To investigate the significance of these differences between the patients and the control group, the *T*-test for independent samples was applied in case of normal distribution. Otherwise, if the new variables were not normally distributed, the Mann-Whitney *U*-test (Tafeit et al., 2003) was used.

**Table 2:** **Effects of orthostatic challenge on thrombelastometry and thrombin generation values**.

	**Baseline samples**	**Post standing samples**	***P*-value**
**THROMBELASTOMETRY**			
**Group 1**			
Coagulation time (CT, s)	319 (178–524)	284 (168–530)	<0.001^a^
Alpha angle (°)	56 (24–72)	60 (23–79)	0.033^a^
**Group 2:**			
Coagulation time (CT, s)	295 (153–726)	283 (115–487)	n.s.^b^
Alpha angle (°)	60 (30–75)	61 (33–75)	n.s.^a^
**CALIBRATED AUTOMATED THROMBOGRAM**			
**Group 1**			
VELINDEX (nM/min)	8 (3–15)	10 (3–34)	0.011^b^
**Group 2**			
VELINDEX (nM/min)	10 (4–17)	11 (3–21)	n.s.^a^
**THROMBIN GENERATION**			
**Group 1**			
Prothrombin fragment 1+2 (pM)	293 (83–740)	387 (122–1000)	<0.001^b^
Thrombin/antithrombin complexes (μg/L)	4.3 (0.9–25.3)	19.7 (1.5–60)	<0.001^b^
**Group 2**			
Prothrombin fragment 1+2 (pM)	266 (167–519)	413 (221–1306)	<0.001^b^
Thrombin/antithrombin complexes (μg/L)	4.0 (1.7–25)	24.4 (3.8–60)	<0.001^b^

*Effects of orthostatic challenge in 22 older patients who had recovered from ischemic stroke (Group 1) compared with 22 healthy age-matched controls (Group 2). Data are expressed as median [interquartile range = Q_1_–Q_3_)]. P-values were calculated by means of the t-test for paired samples (a) in case of normally distributed variables; otherwise the Wilcoxon test (b) was applied*.

## Results

Detailed anthropometric measurements of all test subjects are provided in Table 1. Although, a significantly higher number of patients who had recovered from ischemic stroke were treated with antiplatelet (acetylsalicylic acid and/or clopidogrel) and hypotensive drugs, the basal levels of coagulation parameters were not significantly different between the two groups with the exception of tissue-plasminogen activator. The basal tissue-plasminogen activator levels were significantly higher in the recovered stroke-patients group (10.1 vs. 7.3 ng/mL, medians, *p* = 0.023). Unsurprisingly, the number of previous vascular events and of vascular risk factors was greater in patients when compared with controls.

### Effects of orthostatic challenge on thrombelastometry values

Two thrombelastometry values indicated an orthostatic challenge induced activation of the coagulation parameters in patients but not in healthy controls: CTs were shortened significantly and alpha angles were raised in the post standing whole blood samples (Table 2). The shortening of CTs (difference between standing and baseline) was significantly higher in the stroke patients (Group 1) compared with healthy controls (Group 2), shown in Figure 1. CFT and MCF values were not altered by the orthostatic challenge in either group.

### Effects of orthostatic challenge on CAT values

CAT measurements indicated that orthostatic challenge, in the patients but not in healthy controls, enhances the capability of plasma to generate thrombin. VELINDEX values were significantly increased in the post standing samples in patients- indicating an increased velocity of thrombin formation- compared to baseline samples (Table 2). No significant influence of orthostatic challenge on all other CAT values (Lag time, ETP, Peak, ttPeak, and StartTail) was observed in both groups. Similarly, no significant differences were observed by comparing the coagulation changes between the two groups.

### Effect of orthostatic challenge on levels of F1+2 and TAT

F1+2 and TAT plasma levels indicate that orthostatic challenge is associated with significant thrombin formation in both groups. F1+2 as well as TAT values were markedly increased in the standing samples compared to baseline in patients, and approximately to the same extent in controls (Table 2).

### Effects of orthostatic challenge on standard coagulation times

**Figure 3:**
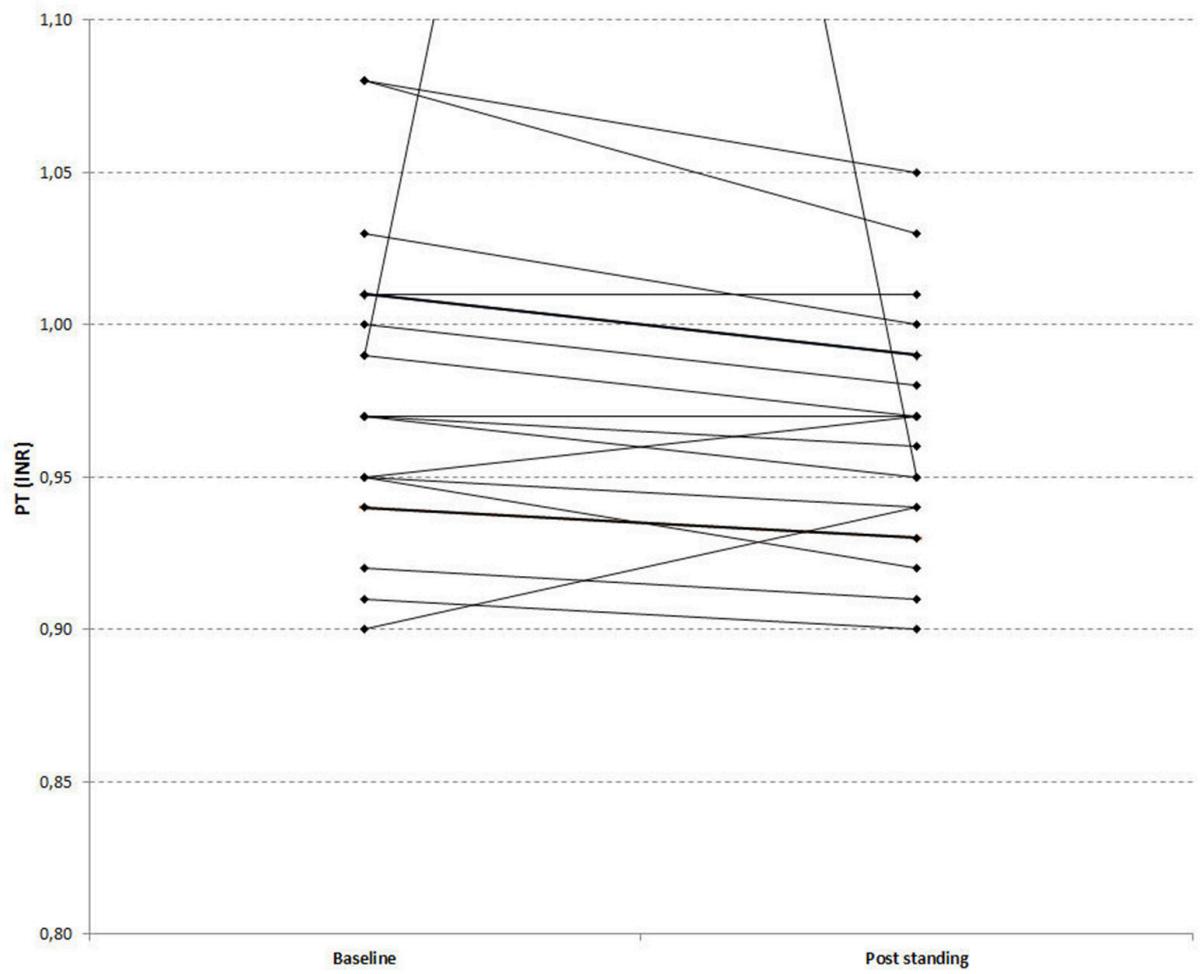
**Effects of orthostatic challenge on Prothrombin Time (INR)**. The figure depicts a paired graph comparing baseline [median (Q1–Q3): 0.98 (0.95–1.02)] and post standing values [median (Q1–Q3): 0.97 (0.94–1.00)] of PT (INR) in stroke recovered patients (*P* = 0.037).

**Figure 4:**
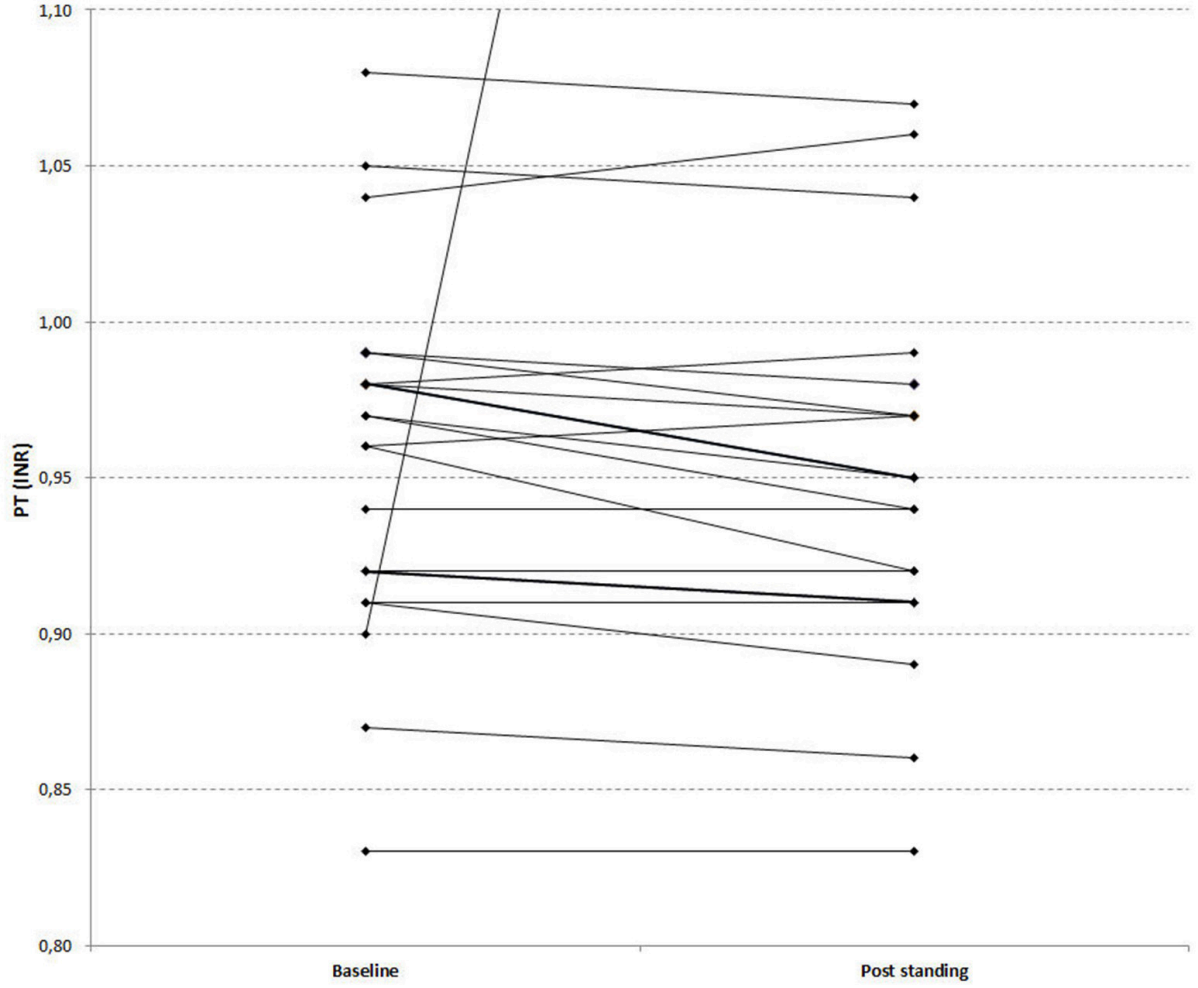
**Effects of orthostatic challenge on Prothrombin Time (INR)**. The figure depicts a paired graph comparing baseline [median (Q_1_–Q_3_): 0.97 (0.92–0.98)] and post standing values [median (Q_1_–Q_3_): 0.95 (0.91–0.98)] of PT (INR) in healthy age matched controls. (*P* = 0.042).

PT values indicated that orthostatic challenge is associated with coagulation activation: PTs (expressed as INR) were significantly shorter in the post challenge samples compared to baseline in both groups. Figure 3 depicts a paired graph showing the PT changes in the stroke recoverd patients and healthy controls (Figures 3, 4; Kimura et al., 2011). APTTs were significantly shortened by orthostatic challenge in healthy controls but not in patients (Table 3). No significant differences were observed by comparing the changes between the two groups.

**Table 3:** **Effects of orthostatic challenge on selected coagulation times and on indicators of endothelial activation**.

	**Baseline samples**	**Post standing samples**	***P*-value**
**STANDARD COAGULATION TIMES**			
**Group 1**			
APTT (s)	36.8 (33.5–38.8)	34.8 (32.3–41.2)	n.s.^b^
**Group 2**			
APTT (s)	37.2 (32.8–39.0)	32.9 (30.8–36.3)	<0.001^a^
**ENDOTHELIAL ACTIVATION**			
**Group 1**			
tPA (ng/mL)	10.1 (8.3–15.5)	9.4 (7.0–15.0)	n.s.^b^
TF (pg/mL)	338 (282–531)	481 (372–591)	0.019^b^
**Group 2**			
tPA (ng/mL)	7.3 (5.5–11.8)	9.7 (6.7–13.7)	0.035^a^
TF (pg/mL)	459 (362–799)	491 (302–1047)	n.s.^b^

*Effects of orthostatic challenge in 22 older patients who had recovered from ischemic stroke (Group 1) compared with 22 healthy age-matched controls (Group 2). Data are expressed as median [interquartile range = (Q_1_–Q_3_)]. P-values were calculated by means of the t-test for paired samples (a) in case of normally distributed variables; otherwise the Wilcoxon test (b) was applied. APTT, activated partial thromboplastin time; TF, tissue factor; tPA, tissue-plasminogen activator*.

### Effects of orthostatic challenge on coagulation factor levels

Orthostatic challenge did not affect FII, FVII, FVIII, protein C and protein S in both groups (data not shown).

### Effects of orthostatic challenge on endothelial activation

Orthostatic challenge appears to cause activation of the endothelium in both patients and healthy controls. Tissue-plasminogen activator plasma values were raised in the standing samples compared to the baseline levels in healthy controls but not in patients with previous ischemic stroke, whereas TF concentrations were increased in the post standing sample in patients but not in the healthy controls (Table 3). Nitrite + nitrate plasma levels were not altered by orthostatic challenge in either group (data not shown). As stated above, the basal levels of tissue-plasminogen activator were significantly higher in the recovered stroke patients group. No significant differences of tissue-plasminogen activator or TF were observed by comparing changes between the two groups.

### Effects of orthostatic challenge on haematocrit/plasma volume

Orthostatic challenge was associated with hemoconcentration in healthy controls but not in patients. The hematocrit was increased in the post standing samples in healthy controls (Table 4). Correspondingly, the percent changes in plasma volume (calculated from hematocrit, according to Masoud et al., 2010) were greater in controls vs. patients (5.34 vs. 2.50%, *p* = 0.002). No significant differences of hematocrit were observed by comparing the changes between the two groups.

**Table 4:** **Effects of orthostatic challenge on haematocrit and on copeptin plasma levels**.

	**Baseline samples**	**Post standing samples**	***P*-value**
**HAEMATOCRIT (%)**			
Group 1	38.1 ± 2.9	38.7 ± 3.4	n.s.^a^
Group 2	38.0 ± 3.0	39.3 ± 3.1	0.002^a^
**COPEPTIN (PG/ML)**			
Group 1	501 (289–748)	627 (308–860)	0.017^a^
Group 2	545 (388–1048)	470 (305–1089)	n.s.^b^

*Effects of orthostatic challenge in 22 older patients who had recovered from ischemic stroke (Group 1) compared with 22 healthy age-matched controls (Group 2). Haematocrit data are presented as mean ± SD, while copeptin data are expressed as median [interquartile range = Q_1_–Q_3_)]. P-values were calculated by means of the t-test for paired samples (a) in case of normally distributed variables; otherwise the Wilcoxon test (b) was applied*.

### Effects of orthostatic challenge on copeptin plasma levels

The response to orthostatic challenge on the hormonal level was significantly higher in patients compared with healthy controls. The copeptin levels were greater in the post standing samples in patients but not in healthy controls (Table 4). The rise of copeptin levels (difference between standing and baseline) was significantly higher in the recovered stroke patients (Group 1) compared with healthy controls (Group 2), shown in Figure 2.

## Discussion

In the present study we quantified coagulation changes in response to orthostatic challenge (a sit-to-stand test) in patients who had recovered from ischemic stroke as compared with healthy, age-matched controls. Although, significantly more stroke-patients were treated with antiplatelet drugs (acetylsalicylic acid and/or clopidogrel), approximately same basal levels of thrombelastometry-derived coagulation times in both groups were found in our study. While acetylsalicylic acid has been shown to possess only a limited capability to affect thrombelastometry values (Trentalange and Walts, 1991; Luddington, 2005), clopidogrel can effectively prolong platelet-fibrin clot formation (Gurbel et al., 2007). Marked effects were found in patients loaded with 300 to 600 mg. We assume that this effect could not be observed in our study since our post-stroke patients were receiving only 75 mg of clopidogrel.

Our data show that orthostatic challenge is associated with activation of the coagulation system. In both groups of test subjects, prothrombin fragment 1+2 as well as thrombin/antithrombin-complex were significantly increased and prothrombin times were significantly shortened in the post standing samples compared to baseline to approximately the same extent. Activated partial thromboplastin times (median) were also shortened due to the orthostatic challenge, reaching significance only in the healthy control group. These findings are in agreement with two other studies, which investigated the effects of still standing on the coagulation cascade (Masoud et al., 2008, 2010). While the results from those studies were based on stationary standing periods that varied from 15 to 60 min, we observed that a short 6 min standing period also leads to a significant activation of the coagulation system. Furthermore, we observed that this activation of the coagulation is significantly more pronounced in patients than in healthy controls. Clotting times, determined by means of thrombelastometry, were significantly shorter in the standing samples compared with baseline in patients recovered from stroke but not in healthy controls. This indicates that orthostatic challenge is associated with a shift toward hypercoagulability in patients who had recovered from ischemic stroke, but not in healthy controls. Accordingly, alpha angles were significantly increased (compared to baseline) by orthostatic challenge in patients but not in healthy controls, indicating that orthostatic challenge apparently leads to enhancement of fibrin built-up and cross-linking in post-stroke patients but not in healthy controls. The changes were approximately the same by comparing both groups.

Moreover, peak rate of thrombin generation values, determined by means of calibrated automated thrombography, were greater in the standing samples in patients but not in healthy controls. This also indicates a shift toward hypercoagulability in patients recovered from stroke: orthostatic challenge seems to be associated with accelerated thrombin formation. Again, the changes were approximately the same when both groups were compared.

Furthermore, we found that orthostatic challenge significantly elevates plasma levels of Tissue Factor (a marker of endothelial activation, Tilley and Mackman, 2006) in patients compared to baseline. This also indicates an orthostatic challenge-induced shift toward hypercoagulability in the stroke-patients group, as Tissue Factor is the most important trigger of the coagulation cascade (Manly et al., 2011). Again, the changes were approximately the same across the two groups.

Plasma levels of tissue-plasminogen activator (another marker of endothelial activation) in healthy controls increased due to orthostatic challenge compared to baseline, indicating increased fibrinolytic activity in the standing samples in this group. However, we found no increase of tissue-plasminogen activator in the patients group compared to baseline, indicating that orthostatic challenge fails to activate the fibrinolytic system in this group (Mazzolai et al., 2002). Again, the changes were approximately the same across both groups.

Levels of another marker of endothelial activation, nitric oxide (determined as nitrite + nitrate plasma levels), were apparently not affected by orthostatic challenge. An explanation might be that the elevated basal levels of nitrite + nitrate in plasma (approximately 60 μM) (Rashid et al., 2003) mask the relatively low amounts of nitric oxide (most likely in the nanomolar range) produced in response to shear stress (Tian et al., 2010).

Interestingly, orthostatic challenge was associated with significant hemoconcentration in healthy controls (~5%) but not in the patient group. Apparently, the microvascular permeability in the stroke-patients group is significantly lower than in the control group. An explanation might be that 73% of the patients, who had recovered from ischemic stroke, but only 32% of the healthy controls, were treated with hypotensive drugs (primarily angiotensin-converting enzyme inhibitors). Angiotensin-converting enzyme inhibitors could have led to the decreases in the microvascular permeability via suppression of angiotensin II formation (Pupilli et al., 1999; Newton et al., 2005). Hematocrit changes were approximately the same when comparing both groups.

Our results also indicate that recovered stroke-patients show more hormonal responses to orthostatic challenge than healthy controls: Plasma levels of copeptin were greater in the standing samples (in comparison to baseline) in patients but not in controls. Copeptin is a valid surrogate marker for arginine-vasopressin release. Due to the arginine-vasopressin being a synergistic component of the hypothalamo-pituitary-adrenal axis, levels of arginine-vasopressin also reflect the participants orthostatic stress response (Katan et al., 2009; Masoud et al., 2010). Copeptin changes were significantly higher in Group 1 compared with Group 2 of test subjects.

We have recently observed that there is some degree of autonomic dysfunction–as reflected in the heart rate variability measurements–during orthostatic challenge even after 1 year of recovery from stroke (Rodriguez et al., in press). As autonomic dysfunction changes have been associated with orthostatic intolerance and clot formation at the site of the vascular injury (Lopez-Vilchez et al., 2009), it is not surprising that a greater tendency toward clot formation was seen in our stroke patients. Our results are also in agreement with the work of Davidoff and colleagues (Davydov et al., 2015).

In conclusion, our data indicate that orthostatic challenge, i.e., a simple sit-to-stand test, leads to a shift toward hypercoagulability in healthy controls, and, to a significantly higher extent, in patients who had recovered from ischemic stroke. In addition to other studies showing coagulation activation after prolonged quiet standing (15 up to 60 min), we show herein that significant coagulation activation occurs even after 6 min of still standing. In our study, protein C plasma levels remained unchanged by orthostatic challenge; this is in agreement with the findings of Masoud et al. (2008, 2010). Thus, it appears that the anticoagulant protein C pathway fails to neutralize the shift toward hypercoagulability (Masoud et al., 2008, 2010).

On the assumption that this simple sit-to-stand test is a valid method that exposes subjects to a procoagulant challenge, our data suggest that the coagulation system of patients with a history of ischemic stroke can be more easily shifted toward a hypercoagulable state than that of healthy controls. Thus, our findings may explain the frequent recurrence of thrombotic events in recovered stroke-patients (Becattini et al., 2012). Our data emphasize the importance of accurate, regular and long-term anticoagulant treatment of patients with a history of thrombosis. It has to be stated that, by absolute numbers, the shift toward a hypercoagulable state in the stroke-patients group is, although statistically significant, relatively small. All coagulation values remained within their reference values, as reported in a previous study (Lamprecht et al., 2013).

Notably, we show herein that even a short-term orthostatic challenge leads to significantly shortened standard coagulation times, particularly Prothrombin Times. With specific regard to the coagulation system, we could not find any literature that deals with the *amount of time* a patient should remain seated before providing blood coagulation assessments. From previous studies it is known that orthostatic challenge induced fluid shifts need about 30 min to return to baseline supine values (Hagan et al., 1978; Hinghofer-Szalkay et al., 2008; Goswami et al., 2012). Furthermore, using head-up-tilt and lower-body-negative pressure (Cvirn et al., 2012), we observed that endothelial activation (as assessed by tissue factor levels) returns to baseline levels around 20 min after orthostatic challenge. As patients are often standing/walking before they reach the consultation room, we recommend that when carrying out assessment of coagulation related parameters, patients should be allowed to rest in a seated or supine position for at least 20–30 min. This will help eliminate the influence of changes in posture on such tests as Prothrombin time.

## What is known on this topic

- Orthostatic challenge causes hypercoagulation.
- Orthostatic challenge induced hypercoagulation has been tested in healthy young subjects.
- Orthostatic challenge induced hypercoagulation has been reported to occur during presyncope induced by head up tilt and graded lower body negative pressure.

## What this paper adds

- Orthostatic challenge causes a significant coagulation activation in patients who have had ischemic stroke more than 1 year prior.
- Orthostatic challenge also shifts the hemostatic system toward hypercoagulation- but to a lesser degree- in healthy older persons.
- Long term anti-coagulant treatment is essential to keep patients from recurrence of vascular events.

## Author contributions

All authors have made significant contributions in data collection, analysis and interpretation of the findings. All co-authors have been involved in writing the final version of the manuscript.

### Conflict of interest statement

The authors declare that the research was conducted in the absence of any commercial or financial relationships that could be construed as a potential conflict of interest.

## Abbreviations

APTT: Activated partial thromboplastin time
ETP: Endogenous thrombin potential
CAT: calibrated automated thrombography
CFT: clot formation time
CT: coagulation time
F 1+2: prothrombin fragment 1+2
INR: international ratio
MCF: maximum clot firmness
PT: prothrombin time
SD: standard deviation
TAT: thrombin/antithrombin-complex
TF: tissue factor
VELINDEX: peak rate of thrombin generation.
